# Questioned validity of Gene Expression Dysregulated Domains in Down's Syndrome

**DOI:** 10.12688/f1000research.6735.1

**Published:** 2015-07-17

**Authors:** Long H. Do, William C. Mobley, Nishant Singhal

**Affiliations:** 1 Department of Neurosciences, University of California, San Diego, CA, 92093, USA

**Keywords:** Gene expression, Down syndrome, iPSC, RNAseq

## Abstract

Recently, in studies examining fibroblasts obtained from the tissues of one set of monozygotic twins (i.e. fetuses derived from the same egg) discordant for trisomy 21 (Down syndrome; DS), Letourneau *et al.,* ^^reported the presence of a defined pattern of dysregulation within specific genomic domains they referred to as Gene Expression Dysregulated Domains (GEDDs). GEDDs were described as alternating segments of increased or decreased gene expression affecting all chromosomes. Strikingly, GEDDs in fibroblasts were largely conserved in induced pluripotent cells (iPSCs) generated from the twin’s fibroblasts as well as in fibroblasts from the Ts65Dn mouse model of DS. Our recent analysis failed to find GEDDs. We reexamined the human iPSCs RNAseq data from Letourneau *et al.*, and data from this same research group published earlier examining iPSCs from the same monozygotic twins. An independent analysis of RNAseq data from Ts65Dn fibroblasts also failed to confirm presence of GEDDs. Our analysis questions the validity of GEDDs in DS.

## Main text

The surprising findings by Letourneau ^1^ and colleagues prompted us to examine our own, as yet unpublished, Ts65Dn transcriptome data for the developing and mature hippocampus in an attempt to identify GEDDs. Our data provided no evidence for the pattern reported in Letourneau *et al.*’s work. We first entertained the possibility that GEDDs were not present in post mitotic cells or cells undergoing neural differentiation. However, to ensure that we fully understood the published GEDD data, we examined the entire RNAseq dataset from the Letourneau manuscript, as provided publicly via the Gene Expression Omnibus. 

Principal component analysis (PCA) of RNAseq replicates from the twin’s fibroblast (T1DS: twin with DS; T2N: disomic twin) revealed a great deal of variability ( Figure 1A). When the datasets from Letourneau *et al.*, are compared, in two of four cases, a closer relationship exists between the DS and disomic twin fibroblasts than for replicates from the same individual. [The datasets from Letourneau *et al.* are denoted by –L]. For example, one of the 2N-hFibro-L replicates clustered more tightly with a DS-hFibro-L replicate than with any of its own replicate (2N) samples.

**Figure 1A.**
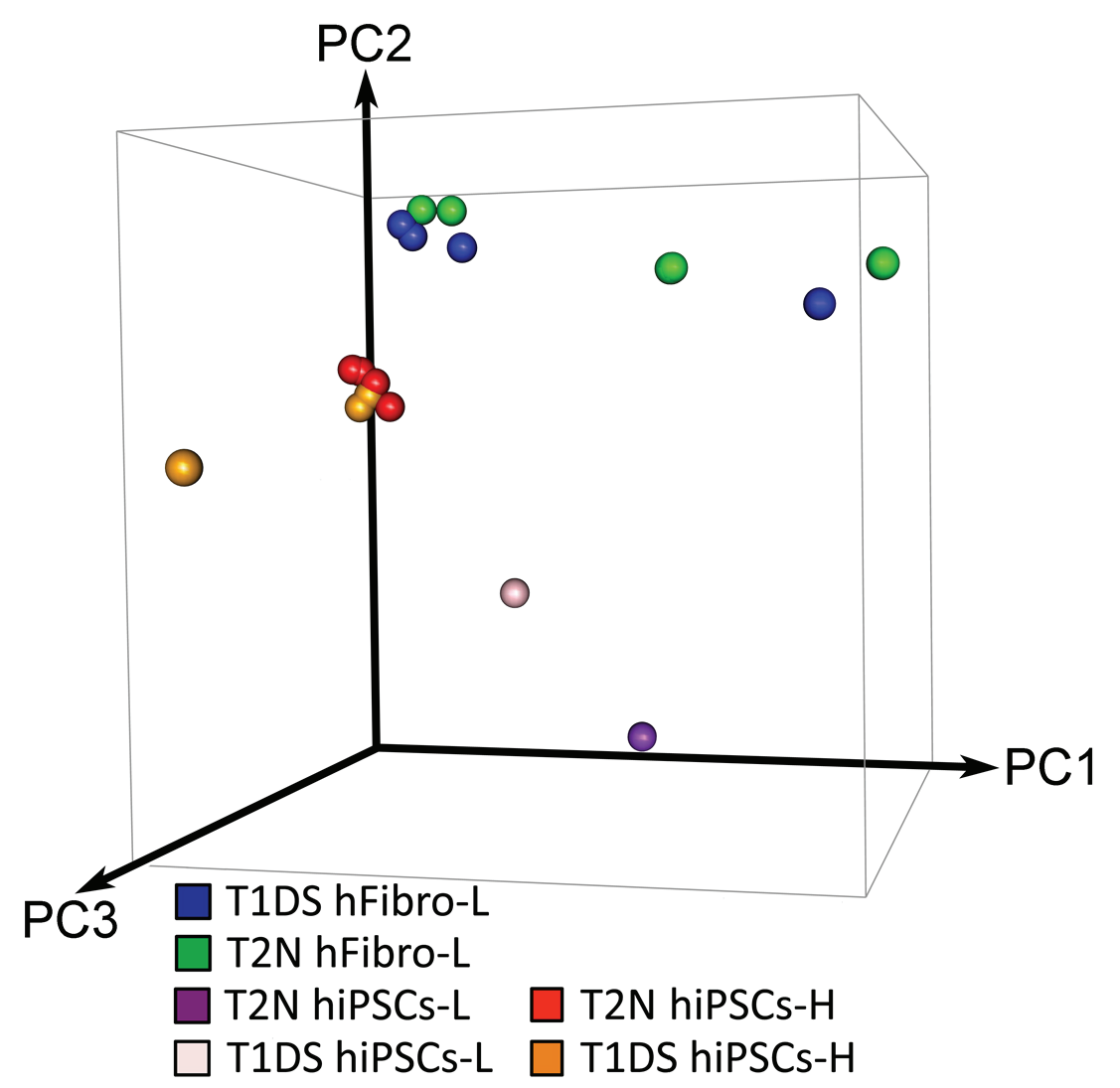
Principal Component Analysis (PCA) of global gene expression (RNAseq) replicates from monozygotic twins discordant for DS. PCA analysis of global gene expression among RNAseq replicates from the twin’s fibroblasts and iPSCs. Comparing hFibro-L replicates with themselves reveals a high degree of variability along the most significant component, PC1. In addition, there is great variability between hiPSCs-L and hiPSCs-H.

We next checked the variability of the twin’s iPSCs RNAseq data. We found an additional three RNASeq replicates (hiPSCs-H) performed by the same research group and published earlier using fibroblasts from the same monozygotic twins ^2^. PCA analysis of these data revealed that replicates of hiPSCs-H (H for Hibaoui) clustered well together; however, they did not cluster well with the data for hiPSCs-L ( Figure 1A). Altogether, PCA analysis indicated marked variability between datasets, raising the possibility that technical issues in the RNAseq samples or in their analysis compromised the Letourneau study.

To further explore the additional three hiPSCs-H RNAseq replicates, we searched for GEDDs using methods similar to those utilized by Letourneau and colleagues. Our analysis of the hiPSCs-H did not find conserved patterns indicative of GEDDs. Figure 1B shows the results for two chromosomes, as examples. The authors reported high global gene fold-change correlations between the twin’s fibroblasts and derived iPSCs. Our re-analysis found a similar high correlation value of ρ = 0.82 between the hiPSCs-L and hFibro-L. However, we found the hiPSCs-H poorly correlated with the original datasets; ρ = 0.31 between hiPSCs-L and hiPSCs-H; ρ = 0.07 between hiPSCs-H and hFibro-L ( Figure 1C and Supplementary figure 1A).

**Figure 1B.**
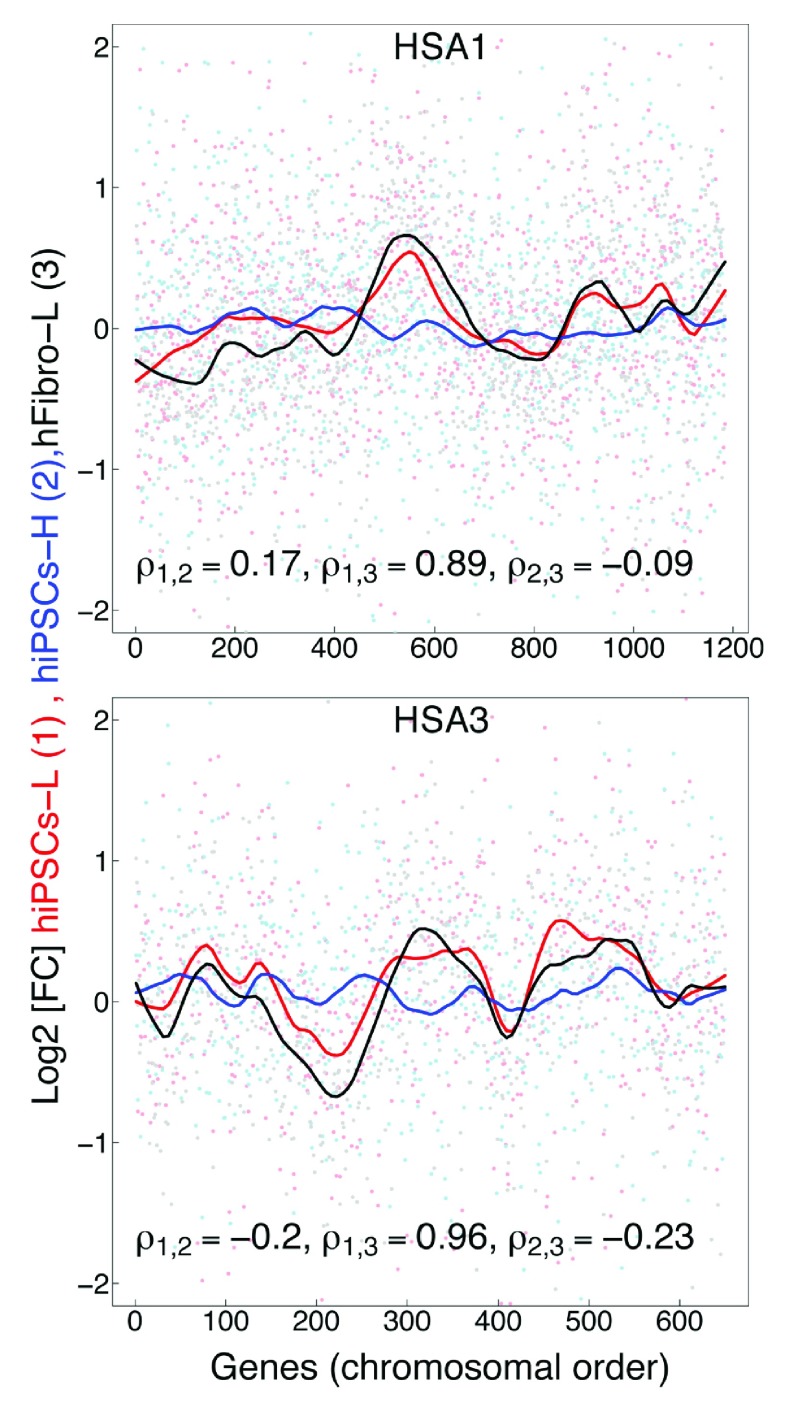
Comparison of the gene expression fold-change profiles between T1DS and T2N in human fibroblasts and human iPS cells along human chromosomes, HSA1 and HSA3. (1) hiPSCs-L derived from monozygotic twins discordant for DS, Letourneau 2014 (red). (2) hiPSCs-H from the same monozygotic twins discordant for DS from Hibaoui 2014 (blue). (3) Human fibroblasts (hFibro-L) from monozygotic twins discordant for DS from Letourneau 2014 (black). hiPSCs-H lack GEDDs, while original hiPSCs-L and hFibro-L show GEDDs and high Spearman’s correlation (ρ _1,3_).

**Figure 1C.**
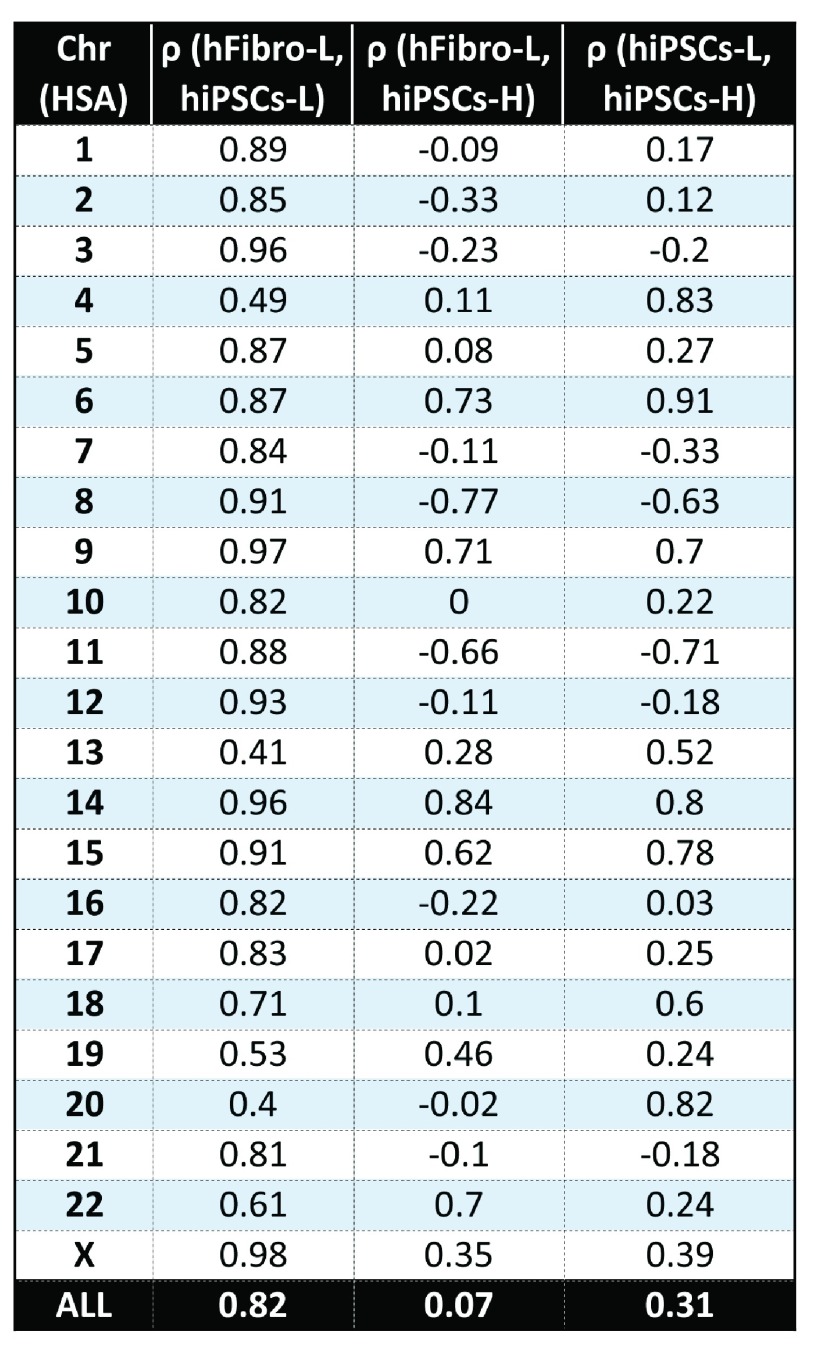
Correlation of global gene expression fold-change profiles from iPSCs and fibroblasts along all human chromosomes. While iPSCs (hiPSCs-L) from the original study are highly similar to fibroblasts (hFibro-L) with a global correlation of ρ=0.82, hiPSCs-H show poor correlation with those samples (ρ=.07, ρ=0.31).

Conservation of GEDDs in Ts65Dn mouse model of DS were quite unexpected given that Ts65Dn mouse is segmentally trisomic (34Mb) for a portion of mouse chromosome 16 (MMU16); the segment contains about 88 mouse homologues to human genes on the long arm of HSA21; it also carries an extra copy of the approximately 10Mb centromeric segment of MMU17 that is not syntenic to any region on human chromosome 21 ^3– 6^. To further explore the possibility of GEDDs, RNAseq data was obtained from three replicates each from Ts65Dn and wild type mouse embryonic fibroblasts (MEFs-D) (D for Do denotes MEFs in the current study). PCA revealed tight clustering between our replicates ( Figure 1D), but not those for the MEFs-L samples. While we found expected changes in gene expression in MEFs-D, analysis of MEFs-L and MEFs-D found a poor global correlation (ρ = -0.31) ( Figure 1E and Supplementary figure 1B); this was also the case across all mouse chromosomes (for examples see Figure 1F and Supplementary figure 1B). In summary our findings raise serious concerns regarding the validity of GEDDs. We find no evidence for such domains in the studies on DS referenced herein or in cells from the Ts65Dn mouse model of DS.

**Figure 1D.**
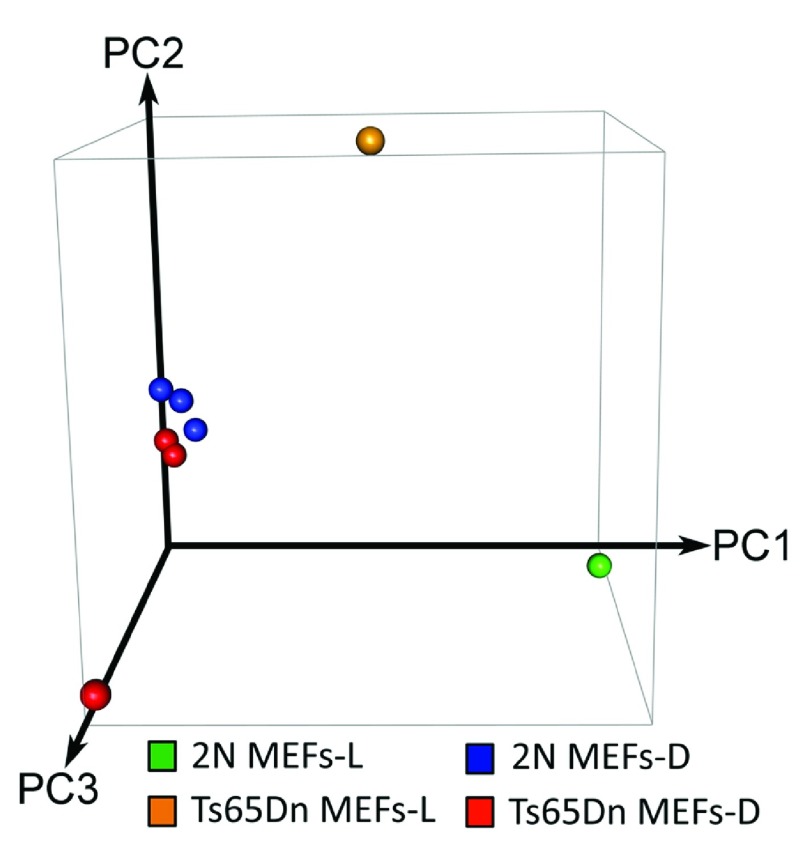
PCA of global gene expression (RNAseq) from normal (2N) and DS model mice (Ts65Dn) embryonic fibroblasts. PCA reveals little variance along the most significant component, PC1, of global gene expression among RNAseq replicates from our repeated experiments, MEFs-D. RNAseq data of mice fibroblasts from the original study, MEFs-L, do not cluster with MEFs-D.

**Figure 1E.**
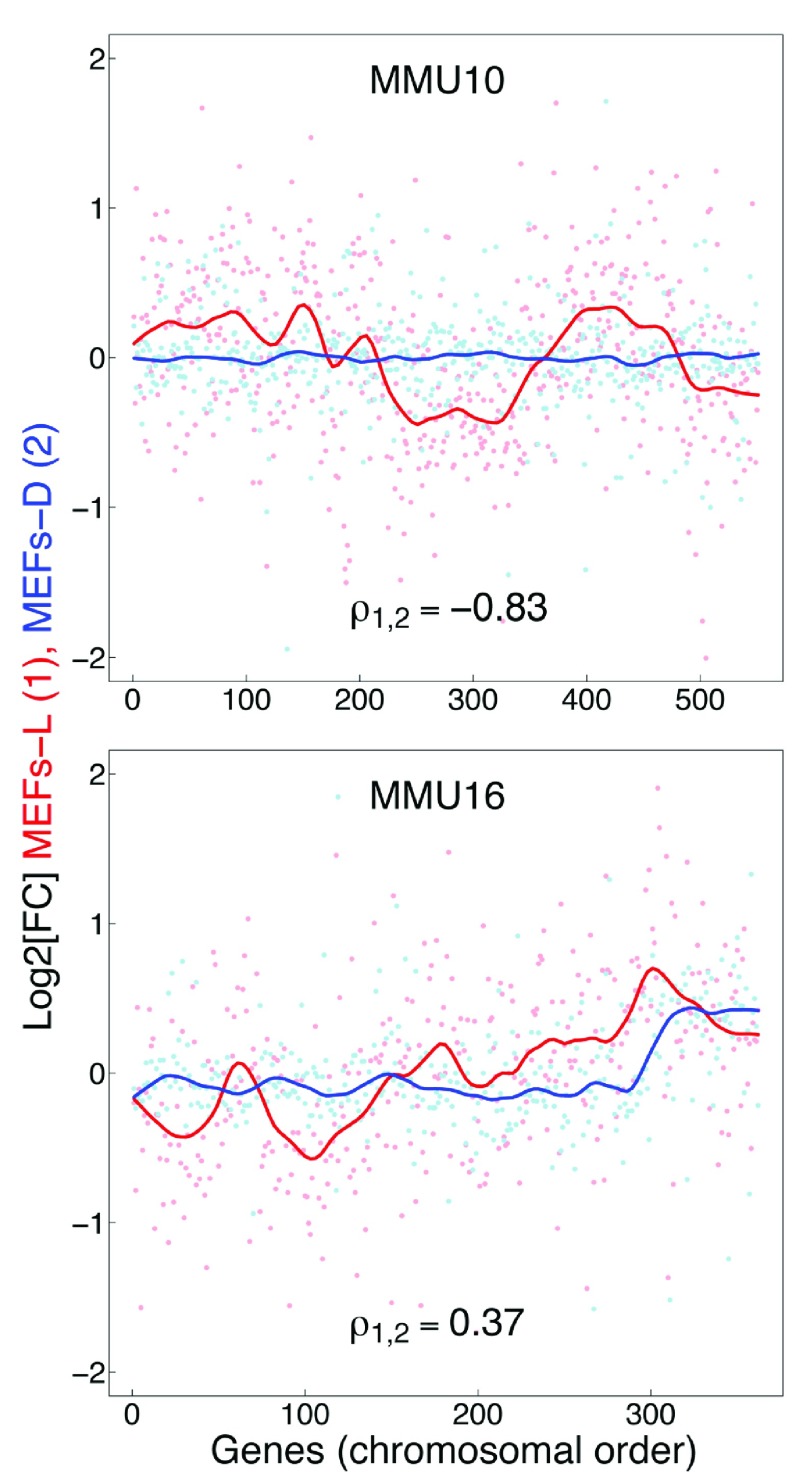
Comparison of the gene expression fold-change profiles between 2N and Ts65Dn fibroblasts plotted with respect to mouse chromosomes 10 and 16. Embryonic mice fibroblasts examined herein (MEFs-D; blue), from 2N and Ts65DN mice do not show the GEDDs reported for MEFs-L (red).

**Figure 1F.**
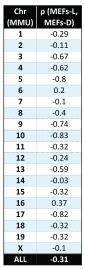
Correlation of global gene expression fold-change profiles from fibroblasts for all mouse chromosomes. Global gene expression fold-change from MEFs-D and MEFs-L show a poor Spearman’s correlation overall (ρ=-0.31) as well as for each of the chromosomes.

## Methods

Total RNA was collected using TRIzol reagent and further purified using RNeasy mini Kit, (Qiagen) from primary mouse embryonic fibroblasts (MEFs) derived from 18.5-day-old Ts65Dn and 2N mouse using manufacturer’s instructions. RNA quality was checked using Tapestation 2200 (Agilent technologies) and quantified using Qubit instrument (Life technologies). TrueSeq stranded mRNA-seq libraries were prepared from 5 μg of total RNA (Illumina mRNA-seq kit, RS-122-2103) and sequenced using Illumina HiSeq 2500 PE-100 (sequences publically available from GEO, accession number: GSE64840). Experiments were performed in triplicate.

RNAseq data from hFibro-L, hiPSCs-L, and hiPSCs-H were downloaded from the Sequence Read Archive (SRP039348, SRP032928) and uploaded to Illumina BaseSpace for mapping (BaseSpace App v1.0, TopHat v2) and differential gene analysis (BaseSpace App v1.1, CuffLinks v2.1.1). PCA was performed using R (v3.1.0) from normalized gene count values (FPKM). Overall Spearman correlation values were calculated from locally weighted scatterplot smoothing (LOWESS) with 30% bandwidth between log2 (FC) gene expression of comparison samples, ordered by genes along each chromosome and plotted using R and custom scripts.

## Software availability

### Software access

Custom scripts for R used to calculate Spearman correlation values are available at https://github.com/lhdo/GEDDplot

### Source code as at the time of publication

https://github.com/F1000Research/GEDDplot/releases/tag/V1

### Archived source code as at the time of publication

http://dx.doi.org/10.5281/zenodo.19232

### Software license

The MIT license

## Supplementary materials

**Supplemental figure S1. A) Comparison of the gene expression fold-change profiles between T1DS and T2N in human fibroblasts and human iPS cells along all human chromosomes.** (1) hiPSCs-L derived from monozygotic twins discordant for DS, Letourneau 2014 (red). (2) hiPSCs-H from the same monozygotic twins discordant for DS from Hibaoui 2014 (blue). (3) Human fibroblasts (hFibro-L) from monozygotic twins discordant for DS from Letourneau 2014 (black). hiPSCs-H lack GEDDs, while original hiPSCs-L and hFibro-L show GEDDs and high Spearman’s correlation (ρ _1,3_). **B) Comparison of the gene expression fold-change profiles between 2N and Ts65Dn fibroblasts along all mouse chromosomes.** Embryonic mice fibroblasts MEFs-D (blue), from 2N and Ts65DN mice do not show the GEDDs reported for MEFs-L (red).

## References

[1] Letourneau A Santoni FA Bonilla X: Domains of genome-wide gene expression dysregulation in Down's syndrome. *Nature.* 2014;508(7496):345–350. 10.1038/nature13200 24740065

[2] Hibaoui Y Grad I Letourneau A: Modelling and rescuing neurodevelopmental defect of Down syndrome using induced pluripotent stem cells from monozygotic twins discordant for trisomy 21. *EMBO Mol Med.* 2014;6(2):259–277. 10.1002/emmm.201302848 24375627 PMC3927959

[3] Busciglio J Capone G O'Bryan J: Down syndrome: genes, model systems, and progress towards pharmacotherapies and clinical trials for cognitive deficits. *Cytogenet Genome Res.* 2013;141(4):260–271. 10.1159/000354306 24008277

[4] Davisson MT Schmidt C Reeves RH: Segmental trisomy as a mouse model for Down syndrome. *Prog Clin Biol Res.* 1993;384:117–133. 8115398

[5] Reinholdt LG Ding Y Gilbert GJ: Molecular characterization of the translocation breakpoints in the Down syndrome mouse model Ts65Dn. *Mamm Genome.* 2011;22(11–12):685–691. 10.1007/s00335-011-9357-z 21953412 PMC3505986

[6] Akeson EC Lambert JP Narayanswami S: Ts65Dn -- localization of the translocation breakpoint and trisomic gene content in a mouse model for Down syndrome. *Cytogenet Cell Genet.* 2001;93(3–4):270–276. 10.1159/000056997 11528125

