# Supplementary material for: Questioned validity of Gene Expression Dysregulated Domains in Down's Syndrome
Source: F1000Res. 2015 Jul 17;4:269. [Version 1] doi: 10.12688/f1000research.6735.1 (PMC4654439; doi:10.12688/f1000research.6735.1)
Supplement: Supplementary file 1 [file f1000research-4-7234-s0000.tgz › b21bd66f-d8f9-4ac5-832f-39920f58f50d.pdf]

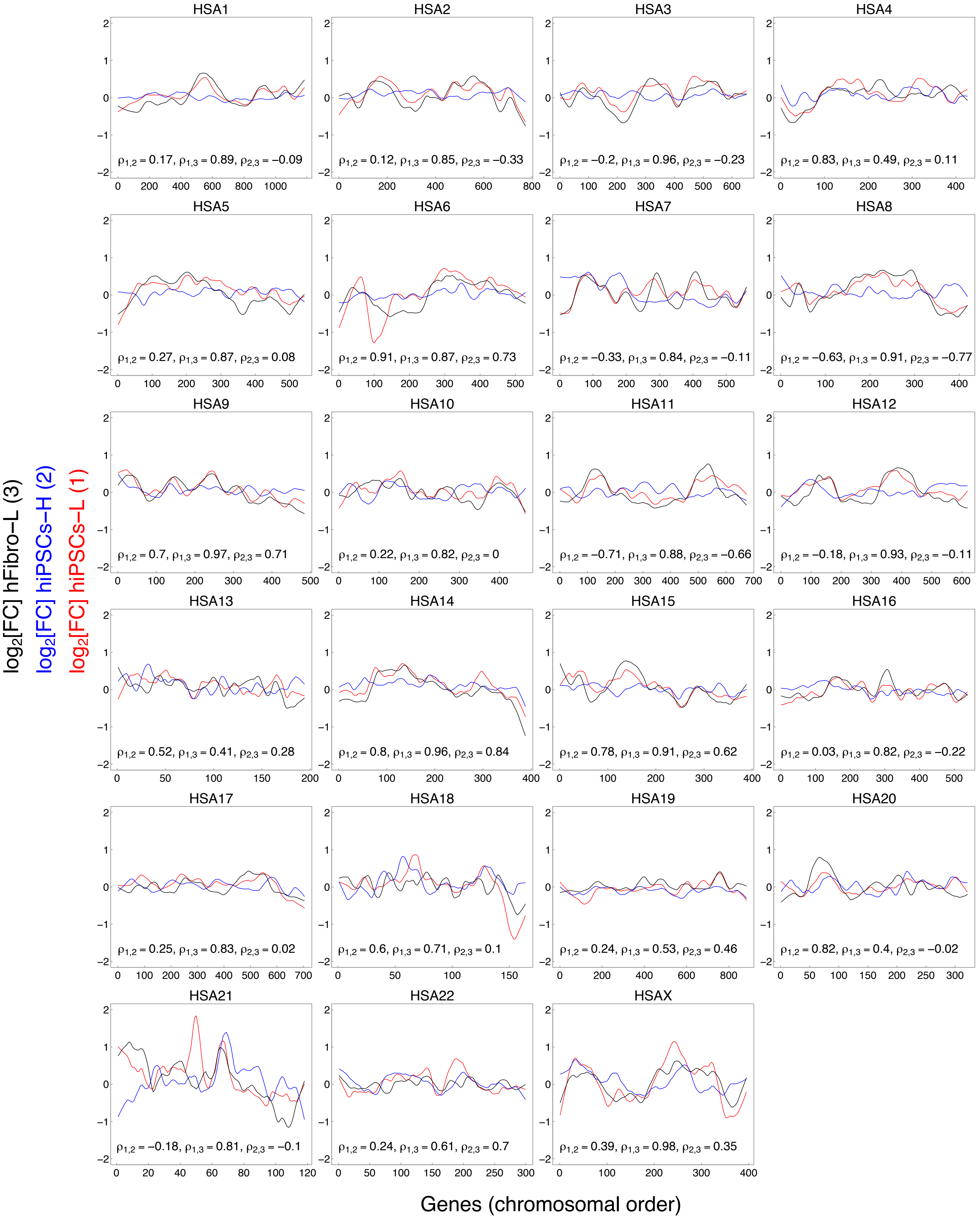

HSA1
ρ1,2 = 0.17, ρ1,3 = 0.89, ρ2,3 = −0.09
HSA2
ρ1,2 = 0.12, ρ1,3 = 0.85, ρ2,3 = −0.33
HSA3
ρ1,2 = −0.2, ρ1,3 = 0.96, ρ2,3 = −0.23
HSA4
ρ1,2 = 0.83, ρ1,3 = 0.49, ρ2,3 = 0.11
HSA5
ρ1,2 = 0.27, ρ1,3 = 0.87, ρ2,3 = 0.08
HSA6
ρ1,2 = 0.91, ρ1,3 = 0.87, ρ2,3 = 0.73
HSA7
ρ1,2 = −0.33, ρ1,3 = 0.84, ρ2,3 = −0.11
HSA8
ρ1,2 = −0.63, ρ1,3 = 0.91, ρ2,3 = −0.77
HSA9
ρ1,2 = 0.7, ρ1,3 = 0.97, ρ2,3 = 0.71
HSA10
ρ1,2 = 0.22, ρ1,3 = 0.82, ρ2,3 = 0
HSA11
ρ1,2 = −0.71, ρ1,3 = 0.88, ρ2,3 = −0.66
HSA12
ρ1,2 = −0.18, ρ1,3 = 0.93, ρ2,3 = −0.11
HSA13
ρ1,2 = 0.52, ρ1,3 = 0.41, ρ2,3 = 0.28
HSA14
ρ1,2 = 0.8, ρ1,3 = 0.96, ρ2,3 = 0.84
HSA15
ρ1,2 = 0.78, ρ1,3 = 0.91, ρ2,3 = 0.62
HSA16
ρ1,2 = 0.03, ρ1,3 = 0.82, ρ2,3 = −0.22
HSA17
ρ1,2 = 0.25, ρ1,3 = 0.83, ρ2,3 = 0.02
HSA18
ρ1,2 = 0.6, ρ1,3 = 0.71, ρ2,3 = 0.1
HSA19
ρ1,2 = 0.24, ρ1,3 = 0.53, ρ2,3 = 0.46
HSA20
ρ1,2 = 0.82, ρ1,3 = 0.4, ρ2,3 = −0.02
HSA21
ρ1,2 = −0.18, ρ1,3 = 0.81, ρ2,3 = −0.1
HSA22
ρ1,2 = 0.24, ρ1,3 = 0.61, ρ2,3 = 0.7
HSAX
ρ1,2 = 0.39, ρ1,3 = 0.98, ρ2,3 = 0.35
log2[FC] hFibro−L (3)
log2[FC] hiPSCs−H (2)
log2[FC] hiPSCs−L (1)
Genes (chromosomal order)

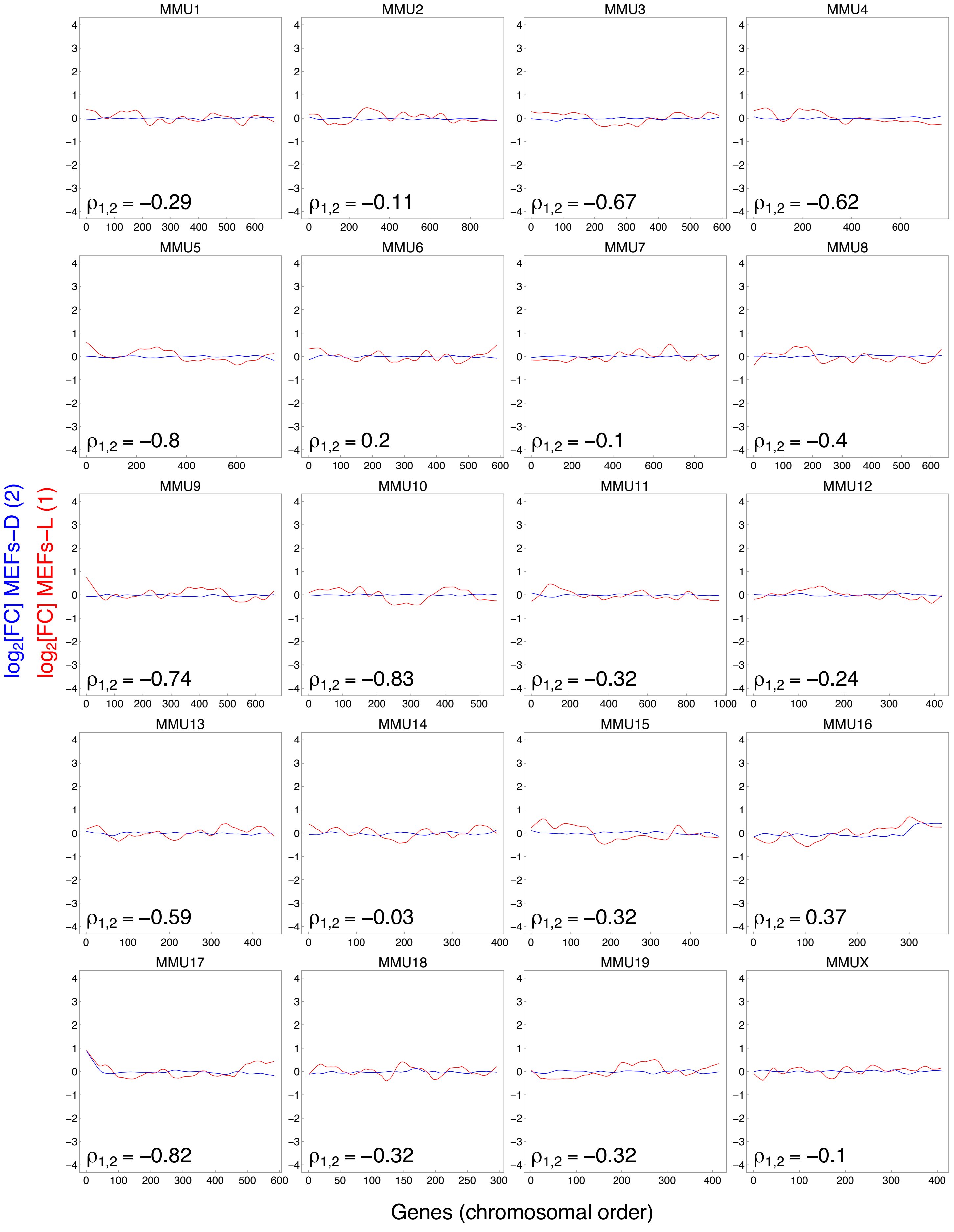
MMU1
ρ1,2 = −0.29
MMU2
ρ1,2 = −0.11
MMU3
ρ1,2 = −0.67
MMU4
ρ1,2 = −0.62
MMU5
ρ1,2 = −0.8
MMU6
ρ1,2 = 0.2
MMU7
ρ1,2 = −0.1
MMU8
ρ1,2 = −0.4
MMU9
ρ1,2 = −0.74
MMU10
ρ1,2 = −0.83
MMU11
ρ1,2 = −0.32
MMU12
ρ1,2 = −0.24
MMU13
ρ1,2 = −0.59
MMU14
ρ1,2 = −0.03
MMU15
ρ1,2 = −0.32
MMU16
ρ1,2 = 0.37
MMU17
ρ1,2 = −0.82
MMU18
ρ1,2 = −0.32
MMU19
ρ1,2 = −0.32
MMUX
ρ1,2 = −0.1
log2[FC] MEFs−D (2)
log2[FC] MEFs−L (1)
Genes (chromosomal order)
