# Supplementary material for: Questioned validity of Gene Expression Dysregulated Domains in Down's Syndrome
Source: F1000Res. 2015 Jul 17;4:269. [Version 1] doi: 10.12688/f1000research.6735.1 (PMC4654439; doi:10.12688/f1000research.6735.1)
Supplement: Peer review history

# Peer review: Referee response for version 1

**DOI:** 10.5256/f1000research.7234.r9542

**Published:** 2015-09-01

**Authors:** Roger Reeves

**Affiliations:** 1 Department of Physiology, School of Medicine, John Hopkins University, Baltimore, MD, USA

Widespread mis-regulation of the expression of disomic genes in trisomic genomes has been established for well over a decade (and seems a self-evident outcome of a trisomy such as that of Hsa21 which includes eight transcription factors, 29 microRNAs and a large number of lncRNAs among other key regulators). Recently, a pattern of chromosome regions showing up- or down- regulation of transcription in the trisomic genome based on RNAseq and substantiated with correlated regional changes in chromosome architecture was reported by Letourneau et al. ( Letourneau et al., 2014). They referred to these regions as “gene expression dysregulated domains” or GEDDs. While the original analysis was carried out in fibroblast and iPS lines derived from a co-isogenic pair of monozygotic fetuses, one of which was euploid and the other of which had trisomy 21, Letourneau et al. report that the pattern of GEDDs is conserved in fibroblasts from the *outbred* Ts65Dn trisomic mouse model of Down syndrome (DS), i.e., the same blocks of disomic genes are up- or down-regulated in a mouse trisomic for orthologs of about half of the genes on Hsa21. These same effects are reported to be undetectable in a pooled comparison of 8 trisomic and 8 euploid cell lines, where they are hypothesized to be masked by normal variability in gene expression expected in individuals who are (obviously) not co-isogenic.

In this report, Do et al. were unable to reproduce these GEDD patterns in an analysis of Ts65Dn mice similar to that which formed a part of the Letourneau evidence. Do et al. then proceeded to compare the Letourneau RNAseq results in this paper to RNAseq results generated previously by this group from cell lines of this same twin pair ( Hibaoui et al., 2014) and found substantial variability in the results obtained in these independently done experiments. Based on several such comparisons in addition to the absence of GEDDS in their independent experiments, Do et al. conclude that there is not clear evidence for the existence of GEDDs.

The comments by Pierce-Shimomura and Nordquist provide an excellent summary of several statistical issues in the analyses. They suggest possible clarifications to the Do study and conclude, with Do, that the existence of GEDDs is not proven. I would note only two additional two points.

1. The PCA analysis in Do et al. comparing datasets from Letourneau and Hibaoui appears to show very strong batch effects, which is in fact the expected outcome for this type of comparison of two studies on different RNA sets prepared and run at different times, probably on different sequencers with different lots of reagents, without reference to placement in sequencing cells, etc. Statistical methods have been developed to clean data for this type of comparison (e.g., Leek JT, Johnson WE, Parker HS, Fertig EJ, Jaffe AE and Storey JD. *sva: Surrogate Variable Analysis*. R package version 3.16.0.). It is not clear that this has been done in the Do analysis.
2. As in all analyses of large platform datasets, it is impossible to determine from the published and supplemental methods what has actually been done at a level of detail required to reproduce it independently – this applies to both studies. (For the exception proving this rule, consider Gilad Y and Mizrahi-Man O. *A reanalysis of mouse ENCODE comparative gene expression data* [v1; ref status: indexed, http://f1000r.es/5ez] *F1000Research* 2015, **4**:121 (doi: 10.12688/f1000research.6536.1) ).

 

However, it is immediately obvious that Letourneau et al. have no basis for many of the statistical comparisons used to identify GEDDs – a *biological* phenomenon - as they have no biological replicates, only technical ones. It was not clear to this reviewer whether Letourneau established four fibroblast clones and/or iPS lines from each twin (and if so, in how many independent transformation experiments) but this would only be a technical replicate for the artifact of that transformation process, not for the biology of GEDDs in trisomy, and therefore it is not the appropriate basis for the statistically-based conclusions that they make about the *biology* of the effects of trisomy 21 on transcription. For biological conclusions, there is an N of one euploid and one trisomic individual – statistical assessments are not possible with a single comparison.

Letourneau et al. argue that genetic variability affecting gene expression levels does not allow detection of GEDDs in any but co-isogenic conditions. However, if GEDDs exist and are so highly conserved in evolution that the same genes are mis-regulated in the same way in outbred* trisomic mice, it is difficult to understand why they are not evident in any comparison of humans with two vs. three copies of Hsa21; if GEDDs only occur in vanishingly rare, coisogenic monozygotic human twin sets discordant for a specific trisomy, the phenomenon would hardly seem worthy of the attention it has received. An explanation for the conservation of this phenomenon in outbred individuals of another species but not between human pairs would strengthen the understanding of this phenomenon. An adequately described experiment comparing multiple individuals with two vs. three copies of Hsa21 including euploid and trisomic sib pairs, in addition to the statistical clarifications suggested by Pierce-Shimomura, would help to establish the existence or not of GEDDs and provide some indication of their relevance to the goal of ameliorating effects of gene dosage in DS.

*Ts65Dn mice are maintained as an advanced intercross between any of several C57BL6 and C3H strains. Thus, individual mice and their sibs are not genetically identical and are heterozygous at ~50% of all loci - a different 50 % for each individual. SNPs occur about every 3000 bp between B6 and C3H, while SNPs for a given segment of a human chromosome pair might be on the order of one per 1000 bp. It might be important to consider variability in chr21 alleles in each twin. Assuming the original conceptus was trisomic, if trisomy resulted from a meiosis I error, the trisomic line will carry three sets of Hsa21 alleles while the euploid twin will lack one of the three sets. If the trisomy resulted from a meiosis II error, the euploid twin could be isodisomic for Chr21 and carry only a single set of alleles. One could speculate about the possible impact of isodisomy on genome-wide expression patterns.

I have read this submission. I believe that I have an appropriate level of expertise to confirm that it is of an acceptable scientific standard.

# Peer review: Referee response for version 1

**DOI:** 10.5256/f1000research.7234.r9541

**Published:** 2015-07-23

**Authors:** Jon Pierce-Shimomura, Sarah Nordquist

**Affiliations:** 1 Department of Neuroscience, College of Natural Sciences, University of Texas at Austin, Austin, TX, USA

Do *et al*. perform statistical analysis on previously published RNAseq data from Letourneau *et al*. (2014) and their own new data to test the prominent and intriguing new hypothesis that trisomy 21 may cause up and down regulation of groups of genes associated with specific physical domains on different human chromosomes (Letourneau *et al*., 2014). These were called gene expression dysregulation domains (GEDDs). Additional evidence suggested that GEDDs may be conserved across human cell types, and surprisingly, may relate to equivalent syntenic domains in the mouse genome after analysis of the Ts65Dn mouse model of Down syndrome (DS) (Letourneau *et al*., 2014).

Here, Do *et al*., first report that replicates from the Letourneau *et al*. (2014) dataset are more variable than one would expect as determined by principle component analysis (PCA). This is an important finding because identification of the purported GEDDs and their extrapolated conservation across tissue types and species depends on minimal variation across datasets. To better understand the significance of the apparent variation in replicate datasets (Figure 1A), it would be useful if Do *et al*. could also plot the percent variance aside each dot accounted for the first principal component. The procedure for data normalization should also be explained more thoroughly in the results section as this may significantly alter the apparent variance by PCA. Do *et al*. also find that data from the Letourneau *et al*., 2014 study varies significantly from data from a previous study by the same lab that used human cells derived from the same source in ( Hibaoui *et al*., 2014).

Do *et al. *then analyze how gene expression changes across physical locations on human chromosomes. They replicate the original finding by Letourneau *et al. *(2014) showing that there are domains of up and down regulated genes from human iPSCS and fibroblasts derived from the same source. However, they also find that these domains fail to correlate with data derived from the same source but published in an earlier study from the same group (Hibaoui *et al*., 2014). This finding raises significant doubt about the concept of conserved GEDDs if they cannot be replicated from tissue derived from the same individual and collected by the same research group. Do *et al*., might do well to suggest explanations for the lack of correlation including specific analysis techniques and methodologies.

Lastly, Do *et al*. find that RNAseq datasets from wild-type and the Ts65Dn mouse model published in the (Letourneau *et al*., 2014) paper show considerable variation from their new set of mouse data as determined by PCA. They also show a lack of correlation between fold-change gene expression for both datasets across chromosomes.

Together, this new analysis suggests a re-evaluation of the GEDDs concept related DS. Specific groups of physically-linked genes (domains) may indeed be up and down regulated in DS across individuals and perhaps in mouse models of DS. However, variation within and across RNAseq datasets appears to prevent defining these domains and generalizing them to other individuals and species with current methods of analysis.

We have read this submission. We believe that we have an appropriate level of expertise to confirm that it is of an acceptable scientific standard.
